# Supplementary material for: Transcriptomic Insights into Mechanisms of Early Seed Maturation in the Garden Pea (Pisum sativum L.)
Source: Cells. 2020 Mar 23;9(3):779. doi: 10.3390/cells9030779 (PMC7140803; doi:10.3390/cells9030779)

Zhewan−1,25(4)

Global Summary

%DE = 0.24

genes with fdr < 0.2 = 490 (234 + / 256 −)

genes with fdr < 0.1 = 425 (209 + / 216 −)

genes with fdr < 0.05 = 393 (196 + / 197 −)

genes with fdr < 0.01 = 327 (168 + / 159 −)

genes in genesets = 0

<FC> = 0

<t−score> = 0

<p−value> = 0.01

<fdr> = 0.76

Portrait

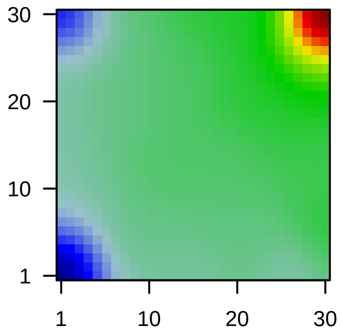

Top 100 DE genes

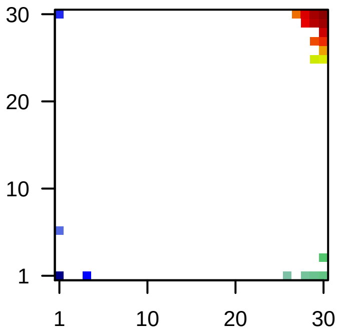

Global Genelist

Rank	ID	log(FC)	p−value	fdr	Metagene	Description
1	TRINITY_DN2	−0.53	2e−16	2e−15	1 x 1	
2	TRINITY_DN1	−0.65	2e−16	2e−15	1 x 1	
3	TRINITY_DN9	0.77	2e−16	2e−15	30 x 30	
4	TRINITY_DN2	1.08	2e−16	2e−15	30 x 30	
5	TRINITY_DN7	−0.98	2e−16	2e−15	30 x 1	
6	TRINITY_DN7	2.62	2e−16	2e−15	30 x 30	
7	TRINITY_DN5	3.65	2e−16	2e−15	30 x 30	
8	TRINITY_DN5	−1.67	2e−16	2e−15	1 x 1	
9	TRINITY_DN5	−0.66	2e−16	2e−15	1 x 30	
10	TRINITY_DN7	1.12	2e−16	2e−15	30 x 30	
11	TRINITY_DN7	−1.28	2e−16	2e−15	1 x 1	
12	TRINITY_DN4	−4.28	2e−16	2e−15	1 x 1	
13	TRINITY_DN4	0.58	2e−16	2e−15	30 x 26	
14	TRINITY_DN9	−0.77	2e−16	2e−15	1 x 1	
15	TRINITY_DN9	1	2e−16	2e−15	30 x 30	
16	TRINITY_DN9	1.98	2e−16	2e−15	30 x 30	
17	TRINITY_DN9	0.6	2e−16	2e−15	27 x 30	
18	TRINITY_DN2	0.55	2e−16	2e−15	29 x 30	
19	TRINITY_DN2	−0.73	2e−16	2e−15	1 x 1	
20	TRINITY_DN2	0.54	2e−16	2e−15	30 x 1	

p−values

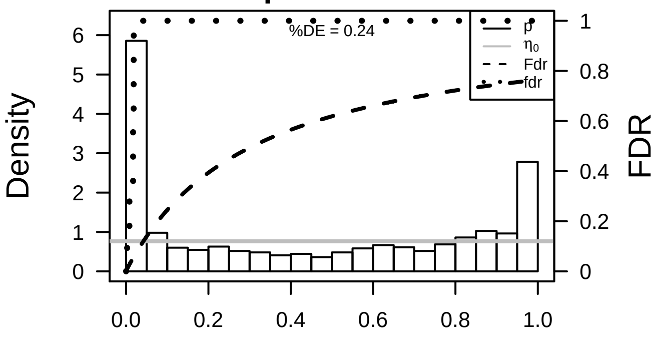

Supplement: Supplementary file 1 [file cells-09-00779-s001.zip › Supplementary materials/FileS4.zip!/FileS3/TFs - Results/Summary Sheets - Samples/Zhewan.1.25.4..pdf]
